# Why should we prioritise smoking cessation for people with mental health conditions?

**DOI:** 10.3399/bjgp23X732921

**Published:** 2023-05-26

**Authors:** Jonathan Campion, Gordon Johnston, David Shiers, Carolyn Chew-Graham

**Affiliations:** South London and Maudsley NHS Foundation Trust, London, UK; Clinical and Strategic Codirector of Public Mental Health Implementation Centre, Royal College of Psychiatrists, London, UK; Public Mental Health Advisor, World Health Organization Europe; Chair of Public Mental Health Working Group, World Psychiatric Association; Honorary Professor of Public Mental Health, University of Cape Town, Cape Town, South Africa.; Person with lived experience of a mental health condition.; Psychosis Research Unit, Greater Manchester Mental Health NHS Trust, Manchester, UK; Honorary Reader in early psychosis, Division of Psychology and Mental Health, University of Manchester, Manchester, UK; Honorary Senior Research Fellow, School of Medicine, Keele University, Keele, UK.; GP, Manchester, UK; Professor of General Practice Research, School of Medicine, Keele University, Keele, UK.

## IMPACT OF SMOKING

Smoking is the single largest cause of preventable death and responsible globally for 7.7 million deaths in 2019.^1^ This is due to increased risk of cardiovascular disease, chronic obstructive pulmonary disease, type 2 diabetes, cancer, and blindness. Smoking is also associated with increased risk of developing mental health conditions (MHCs).^2^^–^^4^ In England, smoking accounted for 74 600 deaths in 2019 and 506 100 hospital admissions in 2019–2020.^5^

People with different types of MHCs are several times more likely to smoke than the general population,^6^^,^^7^ and are responsible for a large proportion of overall tobacco consumption,^8^ with one- third of cigarettes smoked by people with an MHC.^7^ People with MHCs therefore experience disproportionate levels of tobacco- associated harm, which is the single largest contributor to their 7–25- year reduced life expectancy,^9^ and smoking has wider social and economic impacts on this population.

## IMPACT OF SMOKING CESSATION ON MENTAL HEALTH AND EFFECTIVE INTERVENTIONS

**Figure 1.**
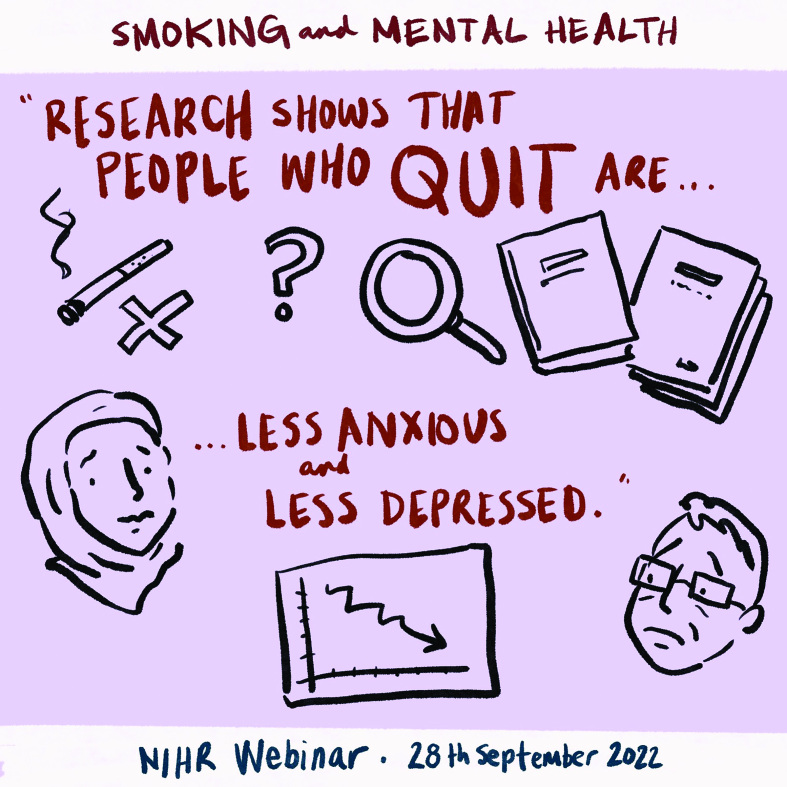
***Illustration capturing discussion at the National Institute for Health and Care Research Three Research Schools webinar, September 2022. Illustration by Tom Bailey. For more illustrations see: https://www.nationalelfservice.net/mental-health/substance-misuse/smokingandmentalhealth-conversations-nihr-3-schools-webinar***.

Smoking cessation results in improved physical and mental health within a few months, including in people with existing MHCs,^10^ as well as financial gains to both the individual and the NHS.^7^ Moreover, smoking cessation is as effective as antidepressants in alleviating mild–moderate depression (Figure 1).^11^ Evidence suggests that people with MHCs are more motivated to stop than those without.^8^

Evidence-based smoking cessation pharmacotherapy and non- pharmacotherapy interventions are effective for people with MHCs.^9^^,^^12^ Comparing pharmacotherapy, combination nicotine replacement therapy (NRT) is equally as effective as varenicline (although varenicline is currently unavailable in the UK).^13^ E-cigarettes are also effective in supporting smoking cessation and less harmful than cigarettes,^14^ with half of UK vapers reporting using e-cigarettes to stop smoking in 2019.^15^

## WHAT DO PRESCRIBERS NEED TO KNOW ABOUT MEDICATION AND SMOKING CESSATION?

Smoking increases metabolism of many psychotropic medications, and on smoking cessation doses of such medications require immediate dose reduction in order to prevent medication toxicity (Box 1). If smoking is resumed, original doses need to be reinstated.

**Box 1.** Required dose changes and monitoring of psychotropic medication following smoking cessation (modified from Taylor *et al*^16^)

**Medication**	**Drugs**	**Required action including medication dose change during smoking cessation**
Antidepressants	Doxepin, duloxetine, fluvoxamine, and trazadone	Monitor closely and dose may require reduction
	Escitalopram	Monitor closely and consider 25% dose reduction
	Mirtazapine	Monitor
	Tricyclic antidepressants	Monitor closely and consider 10%–25% dose reduction over 1 week. Consider further dose reduction

Antipsychotics	Olanzapine	Take plasma level before stopping. On stopping, 25% dose reduction. Repeat plasma level a week after cessation and consider further dose reduction
	Risperidone	Monitor
	Haloperidol	Reduce dose by 25%, monitor carefully, and consider further dose reductions
	Clozapine	Take plasma level before stopping. On stopping, gradual 25% dose reduction over a week. Repeat plasma level a week after cessation and anticipate further dose reduction
	Fluphenazine	Reduce dose by 25%, monitor carefully over 4–8 weeks, and consider further dose reductions
	Zuclopenthixol	Monitor

Mood stabilisers	Carbamazepine	Monitor adverse effects and plasma levels

Benzodiazepines	Diazepam	Monitor closely and consider up to 25% dose reduction over 1 week

## WHY IS THERE A NEED FOR COORDINATED PROVISION OF SMOKING CESSATION INTERVENTIONS?

Smoking cessation interventions can be provided in different settings including primary care,^17^^,^^18^ secondary care,^12^ community pharmacies,^19^ and specialist smoking cessation services. In primary care, it is important to:

- ask about smoking status in consultations with people with MHCs;
- provide smoking cessation advice, including for those not ready to quit;
- monitor or reduce doses of relevant medication (as outlined in Box 1). This requires clear communication and coordination between prescribers and providers of smoking cessation services;
- monitor mental state following smoking cessation; and
- ask about smoking resumption, which requires prompt dose increases of some medications.

Clinicians may wish to use the five A’s model within their consultations (Box 2).^20^

**Box 2.** The five A's model^20^)

**Ask**	Identify and document tobacco use at every clinical encounter
**Advise**	Give person-centred advice about harms of tobacco use and the benefits of quitting
**Assess**	Is the patient willing to consider quitting?
**Assist**	Develop a management plan to support patient to quit
**Arrange**	Proactive follow-up

## WHY IS THE CURRENT EVIDENCE NOT IMPLEMENTED?

In England, only a minority of people with MHCs receive treatment, with far less coverage of interventions to prevent associated impacts such as physical illness and premature mortality.^9^ Smoking cessation is one of the most cost-effective interventions — implementation would save money for the NHS and other sectors.^7^ Despite the impact of smoking and existence of effective smoking cessation and prevention uptake interventions, implementation is poor. For instance, NHS Stop Smoking Services in England supported 1.8% of smokers to successfully quit during 2021/2022.^21^^,^^22^

Primary care has a key role in smoking cessation given smoking has been coded in the primary care records of 32.0% of patients with MHCs compared with 10.9% without in England.^23^ However, cohort studies in England reveal that the proportion of smokers with MHCs receiving NRT fell from 14.4% in 2007 to 3.9% in 2015.^23^ Furthermore, only 8.7% of smokers with depression, 10.1% of smokers with severe mental illness (SMI), and 5.9% of the general population received NRT from primary care, with very- low provision of bupropion or varenicline and <5% of smokers referred to stop smoking services.^24^ Increases in advice over recent years was not accompanied by recorded attempts to quit or smoking status. Primary care prescription items for smoking cessation pharmacotherapies per 100 smokers in England during 2021/2022 were 8.9, including 6.8 for NRT, 1.3 for varenicline, and 0.9 for bupropion.^21^^,^^22^ Over the last 10 years in England, there has been an 81% drop in the number of prescriptions by primary care for smoking cessation pharmacotherapy.^21^

In 2021, 345 402 people with SMI (59.8% of people with SMI) in England had a physical health check that mentioned smoking, although information was unavailable about whether a smoking cessation intervention was delivered in such reviews.^25^

## WHAT IS REQUIRED TO ADDRESS SMOKING IN PEOPLE WITH MHCs?

Several actions are required to reverse the implementation failure of smoking cessation for people with MHCs and support achievement of the English Tobacco Control Plan target that all population groups will reach <5% smoking rates by 2030:^26^

- at local level, integrated care partnerships (ICPs) have a statutory duty to set out how assessed needs, including for smoking cessation and prevention, are to be met by integrated care boards, partner local authorities, and NHS England through the integrated care strategy;
- at national level, UK Government has a responsibility to assess national size of the implementation gap in smoking cessation interventions for people with different MHCs. Transparent agreement with UK Government is then required regarding annual targets for smoking cessation interventions to 2030 to address the gap together with associated resource in order to increase coordinated provision by different settings, including primary care, secondary care, pharmacies, local authority-funded services, and digitally provided interventions;
- systems are required to support data collection and regular monitoring of unmet need, coverage, and outcomes of interventions, including for higher-risk groups by different providers;
- improved skills and knowledge are required for the range of professionals working in primary care, mental health services, pharmacies, and social care; and
- improved population awareness is required about the mental impact of smoking cessation, how to stop smoking, and necessary medication dose changes.

## CONCLUSION

Smoking is the single largest cause of premature mortality in people with MHCs. Primary care should be a key provider of smoking cessation support for people with MHCs — this includes adjusting medication doses to maintain patient safety. Primary care also has an important role to influence strategic decisions to support required coverage of smoking cessation interventions.
